# Mesenchymal Stem Cells Derived from Human Urine-Derived iPSCs Exhibit Low Immunogenicity and Reduced Immunomodulatory Profile

**DOI:** 10.3390/ijms251910394

**Published:** 2024-09-27

**Authors:** Peiyun Wang, Ying Zhang, Zhixing Li, Shenglan Zhou, Qiyu Tang, Zujia Wang, Rou Xiao, Mai Feng, Lingqian Wu, Desheng Liang

**Affiliations:** 1 Center for Medical Genetics & Hunan Key Laboratory of Medical Genetics, School of Life Sciences, Central South University, Changsha 410078, China; wangpeiyun@sklmg.edu.cn (P.W.); zhangying@sklmg.edu.cn (Y.Z.); lizhixing@sklmg.edu.cn (Z.L.); zhoushenglan@sklmg.edu.cn (S.Z.); tangqiyu@sklmg.edu.cn (Q.T.); wangzujia@sklmg.edu.cn (Z.W.); xiaorouyoyo@163.com (R.X.); wulingqian@sklmg.edu.cn (L.W.); 2 Hunan Key Laboratory of Animal Models for Human Diseases, School of Life Sciences, Central South University, Changsha 410078, China; fengmai@sklmg.edu.cn

**Keywords:** induced pluripotent stem cells (iPSCs), mesenchymal stem/stromal cells (MSCs), low immunogenicity, cell therapy, immunomodulation

## Abstract

Human-induced pluripotent stem cell (iPSC)-derived mesenchymal stem cells (iMSCs) represent a promising and renewable cell source for therapeutic applications. A systematic evaluation of the immunological properties and engraftment potential of iMSCs generated from urine-derived iPSCs is lacking, which has impeded their broader application. In this study, we differentiated urine-derived iPSCs into iMSCs and assessed their fundamental MSC characteristics, immunogenicity, immunomodulatory capacity and in vivo engraftment. Compared to umbilical cord-derived MSCs (UCMSCs), iMSCs demonstrated an enhanced proliferative capacity, a higher level of regenerative gene expression, and lower immunogenicity, strengthening resistance to apoptosis induced by allogeneic peripheral blood mononuclear cells (PBMCs) and the NK-92 cell line. In addition, iMSCs exhibited a diminished ability to inhibit T cell proliferation and activation compared with UCMSCs. Transcriptomic analyses further revealed the decreased expression of immune regulatory factors in iMSCs. After transfusion into mouse models, iMSCs engrafted in the lungs, liver, and spleen and exhibited the ability to migrate to tumor tissues. Our results indicated that iMSCs generated from urine-derived iPSCs have a significant replicative capacity, low immunogenicity and unique immunomodulatory properties, and hence offer obvious advantages in immune privilege and allogenic therapeutic application prospects.

## 1. Introduction

Mesenchymal stem cells (MSCs) are multipotent stem cells originating from the early mesoderm, characterized by a high capacity for self-renewal [1]. MSCs exhibit unique immunomodulatory properties and possess the ability to differentiate into osteoblasts, chondrocytes, and adipocytes [2,3]. MSCs are widely employed in the treatment of tissue damage, organ degeneration, aging, and inflammation-mediated diseases in clinical trials [4,5,6,7]. Furthermore, their inherent tumor tropism positions MSCs as highly promising candidates for drug delivery vehicles in cancer therapy [8].

MSCs can be isolated from various tissues, including bone marrow (BMMSCs), adipose tissue (ADMSCs), and the umbilical cord (UCMSCs). Compared to ADMSCs and BMMSCs, UCMSCs exhibit lower immunogenicity, greater proliferative and differentiation potential, slower aging, and stronger anti-inflammatory and immunomodulatory effects, making them increasingly favored for therapeutic applications [9,10,11]. However, MSCs still face many limitations in clinical translation. While they can be readily expanded in vitro, their proliferative capacity is restricted, and they are prone to replicative senescence [12]. Extended in vitro culture and continuous passaging impact their phenotypic characteristics and biological functions [13]. Moreover, the inherent heterogeneity within MSC populations and the variability in quality among different donors present substantial challenges for standardization [14,15].

Induced pluripotent stem cell (iPSC)-derived MSCs (iMSCs) offer a promising solution to these issues of scalability and heterogeneity [16,17]. Urine-derived epithelial cells (urine cells), which can be non-invasively collected from patients of any age or health condition, serve as an ideal source for generating iPSCs (urine-derived iPSCs) [18,19]. Previous research has demonstrated that iMSCs derived from urine-derived iPSCs exhibit surface marker expression, colony-forming capacity, and multi-lineage differentiation potential similar to those of UCMSCs. These iMSCs can be passaged multiple times in vitro while maintaining their MSC characteristics [20,21]. However, comprehensive studies on the immunogenicity, immunomodulatory properties, and in vivo engraftment potential of iMSCs derived from urine cells are lacking, which limits the full understanding of their clinical potential.

In this study, we obtained iMSCs from human iPSCs, which were reprogrammed from urine cells using a safe and non-viral method [22]. Then, the biological characteristics, proliferative capacity, and in vivo biodistribution of iMSCs were assessed. We also compared the immunogenicity and immunomodulatory properties of iMSCs with those of UCMSCs. Our results reveal that, compared to UCMSCs, iMSCs share similar biological features, while exhibiting enhanced proliferative capacity, lower immunogenicity, and reduced immunosuppressive capabilities.

## 2. Results

### 2.1. Differentiation and Characterization of iMSCs

**Figure 1:**
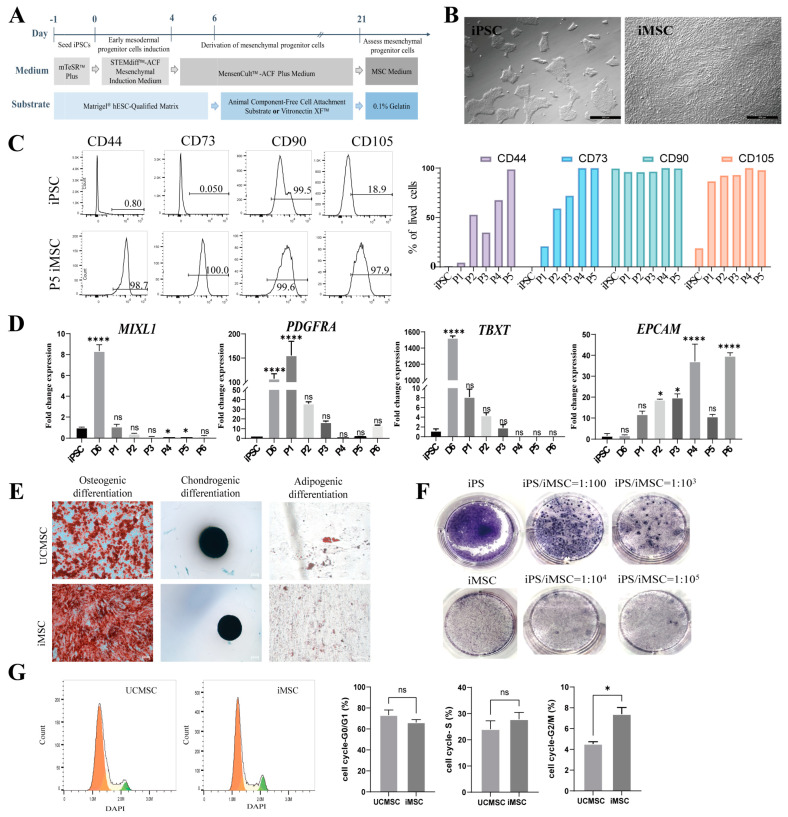
Generation and characterization of iMSCs. (**A**) Schematic representation of the iMSC differentiation protocol. (**B**) Cell morphology of iPSCs and iMSCs at passage 6 (scale bar = 500 µm). (**C**) Flow cytometric analysis of iMSC surface markers CD44, CD73, CD90, and CD105 compared to iPSCs (left). Quantitative assessment of the percentage of positive cells for each marker in iPSCs and iMSCs across passages 1 to 5 (right). (**D**) Relative expression levels of *MIXL1*, *PDGFRA*, *TBXT*, and *EPCAM* in iPSCs and iMSCs (day 6, passages 1–5), as determined by qRT-PCR (mean ± SEM, *n* = 3, one-way ANOVA, ns, not significant, * *p* < 0.05, **** *p* < 0.0001 vs. iPSC). (**E**) Differentiation potential of iMSCs in comparison to UCMSCs. MSCs were induced to differentiate into osteogenic (stained with Alizarin Red, scale bar = 200 µm), chondrogenic (stained with Alcian Blue, scale bar = 200 µm), and adipogenic (stained with Oil Red O, scale bar = 50 µm) lineages. (**F**) Identification of iPSC clones within iMSCs through alkaline phosphatase staining. (**G**) Cell cycle analysis of iMSCs by DAPI staining and flow cytometry (mean ± SEM, *n* = 3, two-sample *t*-test, ns, not significant, * *p* < 0.05).

Human iPSCs were reprogrammed from urine cells by our group, maintaining a normal karyotype and expressing pluripotency markers, including Oct3/4, Nanog, tumor-related antigen (TRA)-1-60, TRA-1-81, and stage-specific embryonic antigen-1 (SSEA-1), as previously described [22]. The iPSCs were differentiated into mesenchymal stem cells (iMSCs) over a 21-day period (Figure 1A and Figure S1A). The iMSCs exhibited a fibroblast-like morphology, distinct from the iPSC clone (Figure 1B). Microscopic imaging further confirmed these morphological characteristics across multiple passages (Figure S1A). Flow cytometry was conducted to assess the expression of surface markers during differentiation from passage 1 to passage 5 (P1–P5). During the expansion phase of mesenchymal progenitor cells (iMSCs P1–P5), two substrates were evaluated: Mesen-Cult-ACF attachment substrate (ACF) and Vitronectin XF™ (VTN). iMSCs cultured on either substrate exhibited similar morphological changes and comparable surface marker expression profiles (Figure S1A,B). Flow cytometry revealed a significant increase in the expression of CD44, CD73, CD90, and CD105 as the cells differentiated from iPSCs to iMSCs (Figure 1C and Figure S1B). Initially, iPSCs were negative for CD44 and CD73, exhibited low expression of CD105, and were positive for CD90. As the cells transitioned to early mesenchymal progenitor cells (P1 iMSCs), CD44 and CD73 expression was initiated, with high levels of CD105. The iMSCs maintained high expression levels of CD105, CD73, and CD90 from P5.

The mesodermal markers *MIXL1* and *TBXT* [23] were significantly upregulated by day 6 of differentiation (Figure 1D). The mesodermal marker *PDGFRA* [24] was markedly upregulated by day 6, peaking at passage P1, but the mRNA levels of these genes declined progressively from P1 to P5, becoming undetectable by passage P6 (Figure 1D). Additionally, *EPCAM* expression increased throughout differentiation, with its encoded protein, EpCAM [25], playing a role in regulating MSC pluripotency and proliferative capacity (Figure 1D). Trilineage differentiation assays demonstrated that iMSCs possess the capability to differentiate into osteocytes, chondrocytes, and adipocytes. Notably, the lipid droplets in UCMSCs were significantly larger than those in iMSCs (Figure 1E). This has also been observed by several other groups [26,27]. The alkaline phosphatase staining of undifferentiated iPSCs resulted in a purple coloration, whereas the differentiated iMSCs remained colorless, indicating the absence of residual iPSCs. The sensitivity of this staining method to detect iPSC contamination at levels as low as 0.001% was confirmed (Figure 1F). The flow cytometric analysis of the cell cycle indicated a significantly higher proportion of iMSCs in the G2/M phase compared to UCMSCs (Figure 1G). iMSCs achieved 80–90% confluency within 4 days following a 1:20 low-ratio cell passage (Figure S1C). These results suggest that iMSCs, derived from iPSCs through mesodermal progenitor cell differentiation, fulfill the criteria for human adult MSCs and exhibit substantial proliferative potential.

### 2.2. Low Immunogenicity of iMSCs

MSCs are characterized by low levels of human leukocyte antigen (HLA) class I and minimal expression of HLA class II, contributing to their low immunogenicity and potential as off-the-shelf therapeutic cells. However, stimulation with inflammatory factors such as Interferon-γ (IFN-γ) or tumor necrosis factor-α (TNF-α) can upregulate HLA expression, potentially eliciting a more robust adaptive immune response [28,29,30,31]. To investigate this, flow cytometry was used to evaluate the surface expression of HLA class I (HLA-A, HLA-B, HLA-C) and class II (HLA-DR, HLA-DP, HLA-DQ) on iMSCs, with or without IFN-γ stimulation.

**Figure 2:**
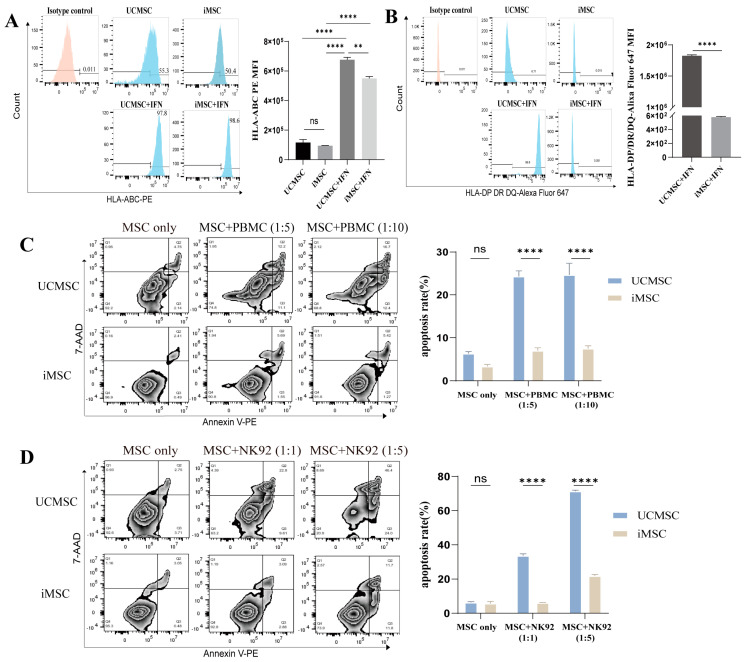
Low immunogenicity of iMSCs. (**A**) Representative histograms showing surface expression of HLA-A/B/C on UCMSCs and iMSCs, with or without Interferon-γ (IFN-γ) stimulation (left), and quantification of HLA-A/B/C mean fluorescence intensity (MFI) (right, mean ± SEM, n = 3, one-way ANOVA, ns, not significant, ** *p* < 0.01, **** *p* < 0.0001 vs. UCMSC). (**B**) Representative histograms of surface expression of HLA-DP/DR/DQ on UCMSCs and iMSCs, with or without IFN-γ stimulation (left), and quantification of HLA-DP/DR/DQ MFI (right, mean ± SEM, n = 3, two-sample *t*-test, **** *p* < 0.0001). (**C**) UCMSCs or iMSCs were co-cultured with PBMCs for 48 h at target-to-effector ratios of 1:5 and 1:10. Apoptosis in UCMSCs or iMSCs was evaluated using Annexin V/7-AAD binding and analyzed by flow cytometry (left). Quantitative analysis of the percentage of apoptotic cells is shown (right, mean ± SEM, n = 3, two-way ANOVA, ns, not significant, **** *p* < 0.0001). (**D**) UCMSCs or iMSCs were co-cultured with NK-92 cells for 24 h at target-to-effector ratios of 1:1 and 1:5. Apoptosis in UCMSCs or iMSCs was assessed using Annexin V/7-AAD binding and analyzed by flow cytometry (left). Quantitative analysis of the percentage of apoptotic cells is shown (right, mean ± SEM, n = 3, two-way ANOVA, ns, not significant, **** *p* < 0.0001).

As shown in Figure 2A, iMSCs and UCMSCs displayed comparable levels of major HLA class I (HLA-A, B, C) expression under basal conditions. After a 24-h culture with IFN-γ (20 ng/mL), the surface expression of HLA class I on both iMSCs and UCMSCs increased (Figure 2A). Under normal culture conditions, neither iMSCs nor UCMSCs expressed significant levels of HLA class II. However, following IFN-γ stimulation, the HLA class II expression increased significantly in UCMSCs, whereas it remained unchanged in iMSCs (Figure 2B).

To evaluate the immunogenicity of iMSCs, allogeneic peripheral blood mononuclear cells (PBMCs) were co-cultured with iMSCs or UCMSCs at various effector-to-target (E:T) ratios, and the apoptosis of MSCs was assessed using Annexin-V/7-AAD staining. Flow cytometric analysis indicated that at E:T ratios of 1:5 and 1:10, iMSCs exhibited higher survival rates compared to UCMSCs due to their lower HLA expression levels (Figure 2C). Additionally, iMSCs were co-cultured with NK-92 cells, an allogeneic natural killer (NK) cell line, to assess susceptibility to NK cell-mediated cytotoxicity. iMSCs were lysed in a dose-dependent manner upon co-culture with NK-92 cells, with significantly lower levels of cell lysis compared to UCMSCs (Figure 2D). These findings suggest that iMSCs exhibit reduced immunogenicity and hold promise as universal cells for therapeutic applications.

### 2.3. Immunomodulatory Capacity of iMSCs In Vitro

**Figure 3:**
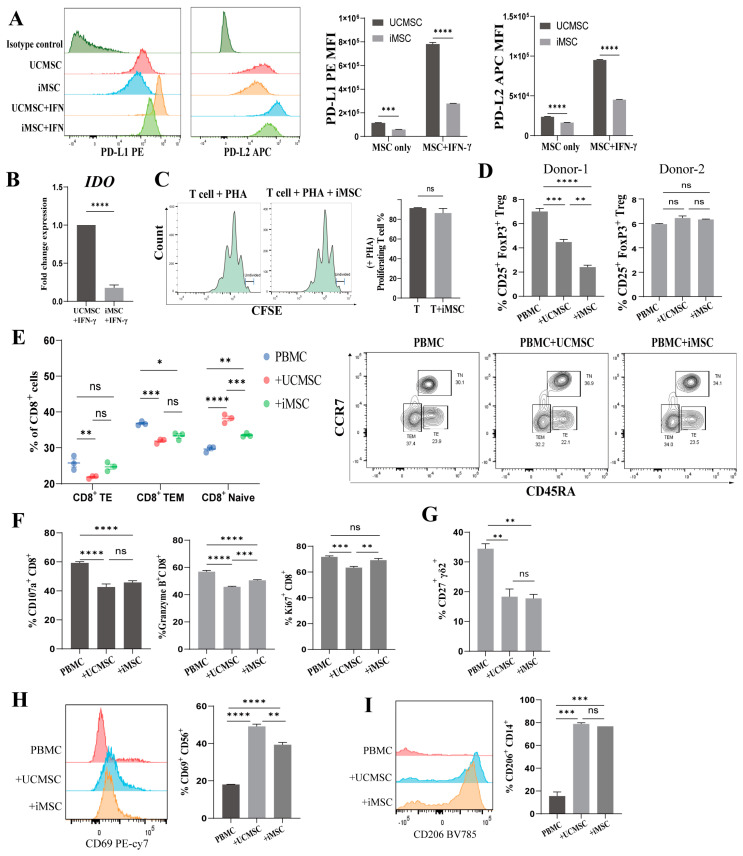
Immunoregulatory effect of iMSCs in vitro. (**A**) Expression of programmed cell death protein ligands 1 (PD-L1) and 2 (PD-L2) in UCMSCs and iMSCs, with or without IFN-γ stimulation, was assessed by flow cytometry (mean ± SEM, n = 3, two-way ANOVA, *** *p* < 0.001, **** *p* < 0.0001). (**B**) Transcription levels of 2,3-polyeneamine dioxygenase (IDO) induced by IFN-γ in UCMSCs and iMSCs were measured by qRT-PCR (mean ± SEM, n = 3, two-sample *t*-test, **** *p* < 0.0001). (**C**) Proliferation of CD3^+^ T cells stimulated by PHA in the presence or absence of iMSCs was tracked using CFSE staining, and the percentage of relative proliferation was analyzed (mean ± SEM, n = 2, ns, not significant, Mann–Whitney test.). (**D**–**I**) The differentiation status, proliferation, and activation of PBMCs and PBMCs co-cultured with UCMSCs (+UCMSC) or iMSCs (+iMSC) were analyzed by flow cytometry. (**D**) Percentages of Treg cells (CD25^+^FoxP3^+^) within CD3^+^CD4^+^ T cell populations in each group (mean ± SEM, n = 3, ns, not significant, ** *p* < 0.01, *** *p* < 0.001, **** *p* < 0.0001). (**E**) Percentages of TE (effector T, CD45RA^+^CCR7^−^), TEM (effector memory T, CD45RA^−^CCR7^−^), and TN (naïve T, CD45RA^+^CCR7^+^) subsets within CD3^+^CD8^+^ T cell populations in each group (mean ± SEM, n = 3, ns, not significant, * *p* < 0.05, ** *p* < 0.01, *** *p* < 0.001, **** *p* < 0.0001). (**F**) Production of CD107a, granzyme B, and proliferation (Ki67^+^) within CD3^+^CD8^+^ T cell populations (mean ± SEM, n = 3, ns, not significant, ** *p* < 0.01, *** *p* < 0.001, **** *p* < 0.0001). (**G**) Analyzed for CD27 marker expression of γδ2^+^ T cell subsets (δ2^+^γ9^+^) (mean ± SEM, n = 3, ns, not significant, ** *p* < 0.01). (**H**) Surface expression of CD69 on NK cells (CD56^+^CD3^−^) (mean ± SEM, n = 3, ** *p* < 0.01, **** *p* < 0.0001). (**I**) Analysis of CD206 expression in macrophage subsets (CD3^−^HLA–DR^+^ CD14^+^) (mean ± SEM, n = 3, ns, not significant, *** *p* < 0.001).

MSCs are widely recognized for their capacity to induce immunosuppression through interactions with both innate and adaptive immune cells. Inflammatory factors such as IFN-γ and TNF-α activate receptors on MSCs, leading to the expression of immunomodulatory molecules, including 2,3-polyeneamine dioxygenase (IDO), prostaglandin E2 (PGE2), and programmed death ligands [32,33]. Following IFN-γ stimulation, both iMSCs and UCMSCs showed an upregulation of PD-L1 and PD-L2 on their surfaces. Notably, the PD-L1 expression was threefold higher and PD-L2 expression twofold higher in UCMSCs compared to iMSCs (Figure 3A). Furthermore, the qRT-PCR analysis demonstrated that IFN-γ stimulation induced IDO expression, with significantly lower IDO mRNA levels in iMSCs compared to UCMSCs (Figure 3B).

To evaluate the regulatory effects of iMSCs on various immune cells in vitro, the Carboxyfluorescein succinimidyl ester (CFSE) dilution method was used to assess the impact of iMSCs on T cell proliferation. Allogeneic T cells were activated with phytohemagglutinin (PHA) and subsequently co-cultured with iMSCs for 96 h. Although a slight reduction in the proportion of proliferating T cells was observed, no significant inhibition was detected (Figure 3C).

The further analysis of T cell subset distribution and activation levels following co-culture with iMSCs revealed no significant alterations in the proportions of CD3^+^CD4^+^ and CD3^+^CD8^+^ T cell populations (Figure S2A,B). An investigation into the potential of iMSCs to induce regulatory T cells (Tregs) revealed a significant reduction in the proportion of CD4+ Tregs in both MSC co-culture groups compared to the PBMC (donor1) control group, with the most pronounced decrease observed in the iMSC co-culture group, which was not observed in the other PBMC (donor2) co-culture group (Figure S2C and Figure 3D).

Compared to the PBMC-only control group, the CD8^+^ T cells in the iMSCs co-culture group exhibited a notable decrease in the effector T (CD45RA^+^CCR7^−^) and effector memory T (CD45RA^−^CCR7^−^) subsets, alongside a significant increase in the naïve T (CD45RA^+^CCR7^+^) subset (Figure 3E). These effects were more pronounced in the UCMSC co-culture group, which further reduced T effector cells and increased T naïve cells. Intracellular flow cytometry was employed to assess the CD8^+^ T cell activation levels. Both UCMSCs and iMSCs were found to inhibit the production of CD107a, granzyme-B, and Ki67 in CD8^+^ T cells (Figure S2D and Figure 3F). Notably, the proportions of granzyme-B^+^ and Ki67^+^ cells were significantly lower in the UCMSC co-culture group compared to the iMSC group. In addition to conventional αβ T cells, MSCs were found to modulate γδ T cells [34]. Vγ9^+^Vδ2^+^ T cells (Vδ2^+^ T cells) are predominantly present in peripheral blood, expressing tumor necrosis factor receptor family member CD27 and secreting pro-inflammatory cytokines such as IFN-γ upon activation [35]. The expression of CD27, the activation marker of Vδ2^+^ T cells, showed a significant decrease after co-culture with iMSCs or UCMSCs.

We further explored the interactions between iMSCs and innate immune cells, specifically natural killer (NK) cells and macrophages, through flow cytometric analysis. Our findings revealed that both iMSCs and UCMSCs upregulated CD69, an early activation marker on NK cells, suggesting that iMSCs possess the capacity to activate NK cells (Figure 3H). Additionally, co-culture with iMSCs led to a significant increase in CD206 expression on CD14^+^ macrophages, indicating that iMSCs may facilitate the reprogramming of macrophages toward an M2 phenotype, similarly to the effects observed with UCMSCs (Figure 3I).

### 2.4. Transcriptomic Analysis of iMSCs and UCMSCs

**Figure 4:**
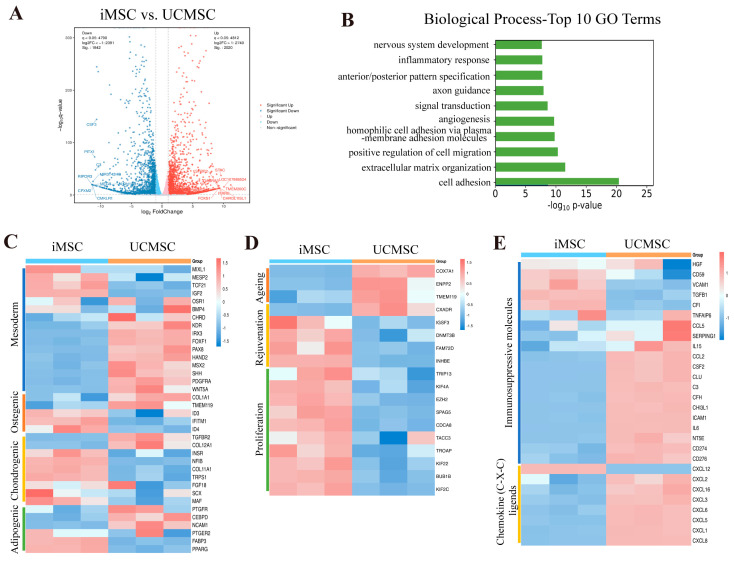
Transcriptomic changes in iMSCs compared with UCMSCs. (**A**) Volcano plot of differentially expressed genes (DEGs) between iMSCs and UCMSCs (q-value < 0.05, |log2 FC| > 1). (**B**) Significantly enriched Gene Ontology (GO) terms related to biological processes in iMSCs versus UCMSCs. (**C**) Heatmap illustrating the expression patterns of selected genes involved in mesoderm differentiation, osteogenesis, chondrogenesis, and adipogenesis in iMSCs and UCMSCs. (**D**) Heatmap depicting the expression patterns of selected genes associated with aging, rejuvenation, and proliferation in iMSCs and UCMSCs. (**E**) Heatmap showing the expression patterns of selected genes involved in immunomodulation in iMSCs and UCMSCs.

We conducted whole-transcriptome analysis to determine the differences between iMSCs and UCMSCs at the transcriptomic level (Figure 4A). Gene ontology analysis revealed that the gene expression changes between iMSCs and UCMSCs were enriched in several biological processes, including the inflammatory response and cell adhesion (Figure 4B). The expression levels of genes associated with mesodermal differentiation [1,27] and trilineage differentiation [36] were also assessed (Figure 4C). iMSCs exhibited a higher expression of genes related to chondrogenesis (e.g., *COL11A1*, *MAF*, *NFIB*, *TRPS1*, *INSR*) and lower expression of genes involved in lipid accumulation (e.g., *PTGFR*, *CEBPD*, *NCAM1*).

Subsequently, we compared the expression of genes related to aging, rejuvenation, and proliferation [37,38,39] between iMSCs and UCMSCs (Figure 4D). iMSCs showed a lower expression of aging signature genes (e.g., *COX7A1*, *ENPP2*, *TMEM119*) and higher expression of rejuvenation-associated genes (e.g., *IGSF3*, *DNMT3B*, *INHBE*) compared to UCMSCs. Additionally, genes associated with proliferation were expressed at higher levels in iMSCs. The expression profile of immune regulatory factors in iMSCs also differed significantly from that of UCMSCs (Figure 4E). Notably, UCMSCs exhibited a higher expression of immunosuppressive molecules and CXCL-chemokines [36,40], indicating that UCMSCs may have a stronger capacity to suppress the proliferation and activation of immune cells (T cells, B cells, NK cells, dendritic cells) and a greater ability to recruit neutrophils and monocytes compared to iMSCs.

### 2.5. Biodistribution of iMSCs in the NCG Mouse Model

**Figure 5:**
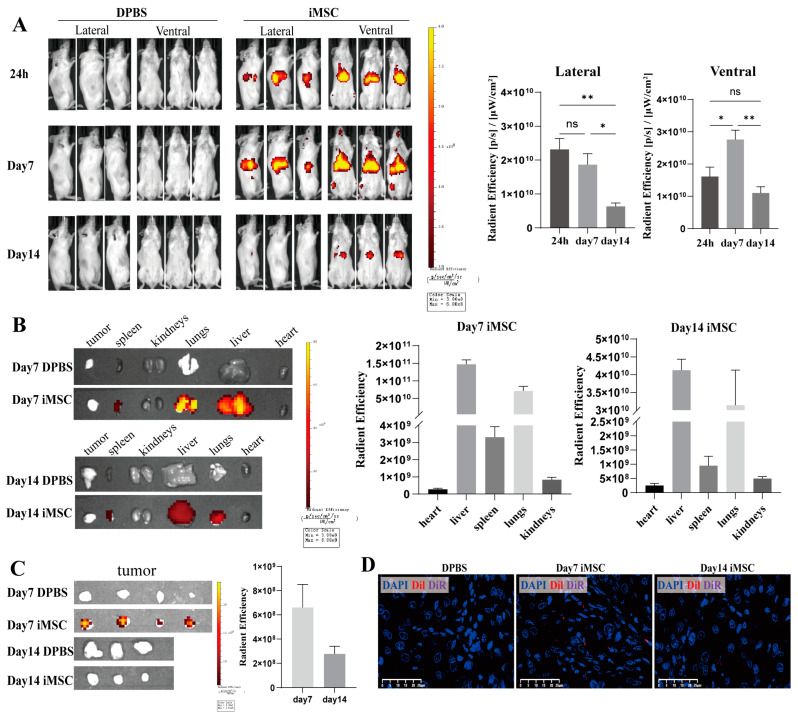
Biodistribution of labeled iMSCs. DiR and DiI double-labeled iMSCs were intravenously injected into NCG mice bearing A549 xenograft tumors. (**A**) In vivo fluorescence of iMSCs was assessed using the IVIS^®^ Imaging System at 24 h, 7 days, and 14 days post-injection (left). Lateral and ventral fluorescence of the mice was quantified using radiant efficiency (right, mean ± SEM, n = 4, one-way ANOVA, ns, not significant, * *p* < 0.05, ** *p* < 0.01). (**B**) Representative ex vivo images of organs captured using the IVIS^®^ Imaging System on days 7 and 14 (left). Fluorescence intensity in various organs was quantified using radiant efficiency (right, n = 4). (**C**) Representative images of tumors obtained using the IVIS^®^ Imaging System on days 7 and 14. Tumor fluorescence was measured by radiant efficiency (right, n = 3–4). (**D**) Representative images of DiI and DiR fluorescent signals in tumor tissue sections detected by immunofluorescence analysis (blue: DAPI, red: DiI, purple: DiR, scale bar = 25 µm).

To investigate the biodistribution and tumor-homing capabilities of iMSCs, we established a xenograft tumor model in highly immunodeficient mice (NOD/ShiLtJGpt-Prkdc^em26Cd52^Il2rg^em26Cd22^/Gpt, NCG) using the A549 lung cancer cell line, with tumors implanted subcutaneously in the right flank. iMSCs labeled with DiR and DiI were injected into the mice through intravenous injection, which is an effective way to deliver MSCs systemically. The distribution of these labeled cells was monitored using IVIS live animal imaging (Figure 5A). Whole-body imaging from lateral and ventral views revealed that iMSCs were detectable after 24 h post-injection, with a notable increase in fluorescence intensity on the ventral side by day 7. By day 14, the fluorescence signal had significantly diminished, with minimal detection of iMSC signals from the lateral view and a threefold reduction in ventral fluorescence compared to day 7 (Figure 5A). On days 7 and 14, organs (heart, liver, lungs, kidneys, and spleen) and tumor tissues were harvested and imaged using the IVIS system to assess the distribution of the labeled iMSCs (Figure 5B,C). The strongest fluorescence signals were observed in the liver on both days 7 and 14, followed by the lungs and spleen, while the signals in other organs were relatively weak. By day 14, the fluorescence signals in all organs had markedly decreased (Figure 5B). In tumor tissues, fluorescence was noted in the iMSC-injected group on day 7, with almost complete disappearance by day 14, compared to the DPBS control group (Figure 5C).

To corroborate the in vivo imaging results, immunofluorescence microscopy was employed to detect the distribution of DiR- and DiI-labeled iMSCs in various mouse tissues. This analysis confirmed the presence of a small number of iMSCs in tumor tissues, with a higher concentration of labeled iMSCs in the liver, spleen, and lungs (Figure 5D and Figure S3). These findings indicate that iMSCs primarily localize to the liver, spleen, and lungs, where they persist for at least 14 days, with a minor proportion homing to tumor tissues. Additionally, we evaluated the acute toxicity of iMSCs following injection (Figure S4A,B). Compared to the DPBS control group, there was no significant weight loss observed in the animals, and blood biochemical parameters remained within normal ranges. These results indicate that iMSC treatment did not induce any MSC-related toxicity.

## 3. Discussion

MSCs are characterized by their multi-lineage differentiation potential and their ability to regulate the immune microenvironment through direct cell contact or paracrine signaling [41,42]. These cells have been extensively investigated for applications in regenerative medicine, disease modeling, and therapy for autoimmune diseases and cancer. Nevertheless, primary MSCs present several challenges, including limitations in cell expansion, considerable population heterogeneity, and difficulties in obtaining enough from patient donors, all of which impede their clinical application [12]. iMSCs have emerged as an attractive and homogeneous source of MSCs. Previous studies have shown that iMSCs possess a morphology, phenotype, and differentiation potential similar to those of primary MSCs, while also demonstrating improved cell viability and proliferative capacity [3,20,37,43]. However, the immunogenicity, immunomodulatory properties, and in vivo engraftment capabilities of iMSCs generated from urine-derived iPSCs, while crucial for their clinical application, are still not quite clear.

We previously collected and reprogrammed urine cells from a healthy donor into iPSCs using a safe and non-viral method [22]. In the current study, the iPSCs were differentiated into iMSCs, and the lower immunogenicity and reduced immunomodulatory capacity of iMSCs compared to UCMSCs were demonstrated. In mouse models, iMSCs predominantly engrafted in the lungs, liver, and spleen following intravenous injection, consistent with findings from previous research [44].

In a previous study, iPSCs were reprogrammed from BM-MSCs and subsequently re-differentiated into iMSCs. These iMSCs failed to inhibit T cell proliferation when co-cultured with T cells [26]. Similarly, in our study, iMSCs did not show significant inhibition of T cell proliferation. Compared to iMSCs, UCMSCs more significantly suppressed the activation of naïve CD8^+^ T cells and their differentiation into CD8^+^ T effector subsets, and reduced CD8^+^ T effector functions. Additionally, both UCMSCs and iMSCs were found to promote NK cell activation, which contrasts with an earlier report indicating that UCMSCs secrete activin A and PGE2 to inhibit NK cell activity [45]. This discrepancy may be attributed to the direct-contact co-culture method used in our study, where NK cells might be affected by the allogeneic immunogenicity of UCMSCs or iMSCs, while the previous study utilized MSC supernatants for co-culturing with NK cells [45].

The findings of this study indicate that the immunomodulatory capabilities of iMSCs differ from those of primary MSCs, potentially due to tissue-specific epigenetic differences of the cells from which the iPSCs are derived. Notably, the gene *TCF21*, which is highly expressed in the kidney [46], was found to be more highly expressed in iMSCs derived from urine cells (Figure 4C), suggesting that iMSCs may retain some degree of epigenetic memory. Previous reports have also highlighted variability in the immunosuppressive abilities of iMSCs depending on their source [26,43,47]. Such variability complicates donor selection and quality control during production, underscoring the importance of developing universal iMSCs.

MSCs are regarded as having immune privilege due to their low expression of HLA class I, absence of HLA class II, and lack of co-stimulatory molecules on their surface. In this study, IFN-γ stimulation led to a significant upregulation of classical HLA-I and HLA-II on the surface of UCMSCs, thereby increasing their immunogenicity in an inflammatory environment and potentially triggering adaptive immune responses [28,29,30,31,48]. To mitigate this, IFN-β and TGF-β were employed to precondition UCMSCs, aiming to reduce their immunogenicity and susceptibility to NK cell-mediated lysis [42]. Previous research has indicated that iMSCs show lower levels of HLA-II upregulation following IFN-γ stimulation compared to primary BM-MSCs [31]. In our study, iMSCs exhibited limited upregulation of HLA-II and displayed superior survival when co-cultured with PBMCs or the NK-92 cell line, compared to UCMSCs, indicating a stronger resistance to cell lysis caused by PBMCs or NK-92 and lower immunogenicity.

However, the reasons for the low immunogenicity and reduced immunomodulatory capacity of iMSCs derived from urine cells remain unclear. We hypothesize that these variations may be attributable to the characteristics of their parental cells. For example, previous studies have demonstrated that iMSCs derived from periodontal ligament cells exhibit a greater capacity for mineralized structure formation [20]. Further research is needed to determine how the immune properties of iMSCs are influenced by the intrinsic properties of the parental cells. Additionally, iMSCs from different donors exhibit varying properties, such as osteogenic differentiation potential [43]. The urine-derived iPSCs used to generate iMSCs in this study were derived from a young, healthy donor. Further research is needed to determine whether donor health or age influences the immune properties of iMSCs.

In this study, iMSCs derived from urine cells demonstrated ease of collection, robust proliferative potential, and high versatility, positioning them as promising candidates for various therapeutic applications. Primary MSCs have been explored as carriers for targeted drug delivery to tumors, with prior research indicating MSC homing to several primary tumor sites, including melanoma, lung cancer, and liver cancer [2,49,50]. However, MSCs can inhibit T cell activation and proliferation, potentially impacting lymphocyte activation within the tumor immune microenvironment. Our study found that iMSCs exhibited in vivo engraftment patterns in animal models and showed tumor tropism, akin to those of primary MSCs [42,49,51]. Furthermore, the advantages of using iMSCs in cancer therapy include their reduced negative impact on immune cells and lower immunogenicity as found in the present study, which contribute to better survival and prolonged drug delivery in vivo. Moreover, the low immunogenicity of iMSCs reduces the risk of immune-related adverse reactions, thereby enhancing the safety profile of iMSC-based therapies. However, in the context of inflammatory or immune-related diseases, such as diabetes type I, Crohn’s disease, and allograft-related diseases [41], the therapeutic efficacy of iMSCs may be constrained by their diminished immunosuppressive capacity.

In summary, this study successfully generated iMSCs from human urine-derived iPSCs, and these cells demonstrate stable MSC characteristics, in vivo engraftment capabilities and superior proliferative and regenerative properties. Additionally, compared to UCMSCs, these iMSCs exhibited lower immunogenicity and a unique immunomodulatory capacity. Our findings indicate the potential of iMSCs generated from urine-derived iPSCs as a safe and universal cell resource for allogenic therapeutic application.

## 4. Materials and Methods

### 4.1. Cell Culture

Human iPSCs were reprogrammed from human urine cells as previously described [22]. The iPSCs were cultured on Matrigel (354277, Corning, Corning, NY, USA) in mTeSR1 medium (85850, STEMCELL Technologies, Vancouver, BC, Canada). The iMSCs were subsequently derived from these iPSCs. Human UCMSCs were obtained from Yuanpin Cell Biotechnology Group Co., Ltd. (Changsha, China). All MSCs were cultured on 0.1% gelatin-coated dishes in MSC medium, which consisted of Minimum Essential Medium α (12571063; Thermo Fisher Scientific, Dreieich, Germany), 10% fetal bovine serum (Gibco, Carlsbad, CA, USA), 100 U/mL L-glutamine (25030081, Gibco, Grand Island, NY, USA), and 10 µg/mL basic fibroblast growth factor (GF003, Sigma-Aldrich, St. Louis, MO, USA).

Fresh PBMCs were isolated from the peripheral blood of healthy donors using Histopaque^®^-10771 (10771, Sigma-Aldrich, St. Louis, MO, USA) via density gradient centrifugation. CD3^+^ T lymphocytes were subsequently isolated from the PBMCs using CD3 microbeads (130-097-043, Miltenyi Biotec, Cologne, Germany). Both PBMCs and CD3^+^ T cells were cultured in RPMI 1640 medium supplemented with 10% fetal bovine serum (FBS). Natural Killer-92 (NK-92) cell lines were obtained from our laboratory’s cell bank and cultured using an NK-92 Medium Kit (TBDNK92KIT, TBD Science, Tianjin, China). A549 cells were purchased from the American Type Culture Collection (ATCC, Manassas, VA, USA) and cultured in high-glucose DMEM (11965092, Gibco) supplemented with 10% FBS.

### 4.2. Human iPSC Culture and Differentiation

iMSCs were derived from induced pluripotent stem cells (iPSCs) using the STEMdiff Mesenchymal Progenitor Kit (05240, STEMCELL Technologies, Cambridge, MA, USA) in accordance with the manufacturer’s protocol. Briefly, iPSCs at 30–40% confluency were transitioned from mTeSR1 medium to mesenchymal induction medium, with daily medium changes for 4 days. Following this induction phase, the medium was replaced with MesenCult-ACF medium for an additional 2 days. Once the differentiated cells reached 80–90% confluency, they were passaged onto plates pre-coated with either MesenCult-ACF attachment substrate or Vitronectin XF™ (07180, STEMCELL Technologies), and cultured in MesenCult-ACF medium. MSC-like cells were maintained through passage 5. Subsequently, iMSCs were cultured on 0.1% gelatin-coated plates in MSC medium (Figure 1A and Figure S1A).

### 4.3. Characterization of MSC Surface Markers Using Flow Cytometry

During the differentiation of iPSCs to iMSCs, cells at passages 1 through 5 were stained with anti-CD73/PerCP-Cy5.5, anti-CD44/FITC, anti-CD90/PE-Cy7, anti-CD105/APC, anti-CD45/Pacific Blue, anti-CD34/Pacific Blue, and anti-HLA-DR/Pacific Blue (all from BD Biosciences, Franklin Lakes, NJ, USA). The stained cells were then analyzed using a BD flow cytometer.

### 4.4. Osteogenic, Chondrogenic and Adipogenic Differentiation of MSCs

The osteogenic, chondrogenic, and adipogenic differentiation of UCMSCs and iMSCs (passage 6) was conducted using the Human Osteogenic Differentiation Kit (05465, STEMCELL Technologies, Cambridge, MA, USA), Human Chondrogenic Differentiation Kit (05455, STEMCELL Technologies), and Human Adipogenic Differentiation Kit (05412, STEMCELL Technologies). UCMSCs and iMSCs were seeded and cultured according to the manufacturer’s protocols. After 2 weeks, the osteogenic and chondrogenic differentiation was evaluated using Alizarin Red (ALIR-10001, Ori Cell, Guangzhou, China) and Alcian Blue (ALIR-10001, Ori Cell) staining, respectively. Adipogenic differentiation was assessed after 3–4 weeks using Oil Red O staining (OILR-10001, Ori Cell).

### 4.5. Alkaline Phosphatase Staining

iMSCs were co-seeded with iPSCs in a 12-well plate at various ratios (100:1, 10^3^:1, 10^4^:1, 10^5^:1). The mixed cells were cultured in MSC medium and stained for alkaline phosphatase (ALP) on day 9 using the Alkaline Phosphatase Stain Kit (40749ES60, Yeasen, Shanghai, China).

### 4.6. Real-Time Quantitative Polymerase Chain Reaction (RT-qPCR) Analysis

mRNA was isolated from hiPSCs and MSCs using TRIzol reagent (Sigma), and quantified with the NanoDrop 2000 (Thermo Fisher Scientific, Waltham, MA, USA). Then, 0.5 µg RNA sample was converted to cDNA using the HiScript II- cDNA Synthesis Kit (R211, Vazyme, China). Following 5-fold dilution of cDNA, RT-qPCR was performed on a Bio-Rad CFX96 Real-Time System using the ChamQ Universal SYBR qPCR Master Mix (Q711, Vazyme). The *ACTB* gene was used as an internal control to normalize relative gene expression. The threshold cycle (Ct) value for each gene was normalized to that of *ACTB*, and relative expression levels were calculated via the ΔΔCT method. The sequences of the primers are as follows:

*MIXL1*-Forward: 5′GGCGTCAGAGTGGGAAATCC3′;
*MIXL1*-Reverse: 5′GGCAGGCAGTTCACATCTACC3′;
*TBXT*-Forward:5′CTATTCTGACAACTCACCTGCAT3′;
*TBXT*-Reverse:5′ACAGGCTGGGGTACTGACT3′;
*PDGFRA*-Forward:5′TGGCAGTACCCCATGTCTGAA3′;
*PDGFRA*-Reverse: 5′CCAAGACCGTCACAAAAAGGC3′;
*EPCAM*-Forward:5′AATCGTCAATGCCAGTGTACTT3′;
*EPCAM*-Reverse:5′TCTCATCGCAGTCAGGATCATAA3′;
*IDO*-Forward:5′TCTCATTTCGTGATGGAGACTGC3′;
*IDO*-Reverse: 5′GTGTCCCGTTCTTGCATTTGC3′;
*ACTB*-Forward:5′TGGTGGGTATGGGTCAGAAGGACTC 3′;
*ACTB*-Reverse: 5′ CATGGCTGGGGTGTTGAAGGTCTCA 3′.

### 4.7. Cell Cycle Analysis

UCMSCs (passage 6) and iMSCs (passage 13) were fixed with 70% ethanol at −20 °C overnight. The fixed MSCs were then stained with DAPI (D1306, Sigma Aldrich, Burlington, MA, USA), a fluorescent dye, and subsequently analyzed using a Cytek^®^ Northern Lights cytometer (Cytek Biosciences, Fremont, CA, USA).

### 4.8. MSC Apoptosis Analysis Using Flow Cytometry

UCMSCs and iMSCs were labeled with CellTrace™ Far Red (C34572, Thermo Fisher Scientific, Waltham, MA, USA) according to the manufacturer’s protocol. Subsequently, 1 × 10^5^ MSCs were co-cultured with 5 × 10^5^ or 1 × 10^6^ PBMCs for 48 h, or with 1 × 10^5^ or 5 × 10^5^ NK-92 cells for 24 h, at 37 °C in 5% CO_2_. Apoptotic MSCs, labeled with CellTrace™ Far Red, were quantified using the Annexin V/7-AAD Kit (559763, BD Biosciences, Franklin Lakes, NJ, USA) and analyzed on a Cytek^®^ Northern Lights cytometer.

### 4.9. Carboxyfluorescein Succinimidyl Ester (CFSE) Proliferation Assay

Here, 2.5 × 10^5^ CD3^+^ T cells, labeled with the CFSE Cell Division Tracker Kit (423801, BioLegend, San Diego, CA, USA), were cultured with 5 × 10^4^ MSCs using 0.4 μm Transwell inserts (3460, Corning, Corning, NY, USA). To activate the T cells, 4 μg/mL phytohaemagglutinin (PHA; 40110ES08, Yeasen, Shanghai, China) was added. After 96 h, the T cells were collected and analyzed using a Cytek^®^ Northern Lights cytometer.

### 4.10. Assessment of PBMC Subsets and Activation

Fresh human PBMCs were labeled with CellTrace™ Violet (Catalog #C34571, Thermo) and then co-cultured with UCMSCs or iMSCs for 72 h. Following co-culture, the PBMCs were collected, resuspended at 1 × 10^6^ cells/100 μL, and stained with the following surface markers (all from BioLegend): CD45/PE-Cy5, CD3/BV505, CD4/AF700, CD8/BV750, CD25/PE, FoxP3/BV421, CD56/BV510, CD45RA/Spark Blue 550, CCR7/PE-Fire 810, CD69/PE-Cy7, CD14/BV711, HLA-DR/APC-Cy7, CD206/BV78, CD27/BV570, TCR γδ/PE-Fire 810, TCR Vδ2/PE-Cy7, TCR Vγ9/PE and Zombie NIR Fixable Viability Kit. Intracellular staining was performed with CD107a/APC-Cy7, Granzyme B/FITC, and Ki67/BV650 according to the manufacturer’s protocols. PBMCs were then analyzed using a Cytek^®^ Northern Lights cytometer.

### 4.11. MSC Flow Cytometry

UCMSCs or iMSCs were stimulated with 20 ng/mL IFN-γ (285-IF-100, R&D Systems, Minneapolis, MN, USA) for 24 h. Following stimulation, the cells were stained with the following cell surface markers: HLA-ABC/PE (311405, BioLegend), HLA-DR DP DQ/AF647 (563591, BD Biosciences), PD-L1/PE (329705, BioLegend), and PD-L2/APC (329608, BioLegend) for 30 min in the dark. The stained cells were then analyzed using a flow cytometer.

### 4.12. Animal Experiments

All animal studies were approved by the Animal Ethics and Experimentation Committee of the School of Life Sciences, Central South University. Six-week-old female NCG mice (NOD/ShiLtJGpt-Prkdc^em26Cd52^Il2rg^em26Cd22^/Gpt) were purchased from GemPharmatech Co., Ltd. (certificate no. SCXK [Su] 2023-0009; Nanjing, China). The mice were housed in a specific-pathogen-free (SPF) facility under a 12-h light/dark cycle and had ad libitum access to food and water. After a 1-week acclimation period, 4 × 10^6^ A549 cells were subcutaneously injected into the right flank of each mouse. Ten days post-injection, the mice were randomly divided into two groups, and each group included 8 mice. The mice were treated with DPBS (150 μL/mouse) or labeled iMSCs (2 × 10^6^ cells in 150 μL DPBS/mouse).

To evaluate the biodistribution of systemically administered iMSCs using IVIS imaging and immunofluorescence, the mice were intravenously injected with 2 × 10^6^ iMSCs double-labeled with DiR (125964, Revvity, Waltham, MA, USA) and DiI (C7000, Invitrogen, Waltham, MA, USA). The biodistribution of iMSCs was monitored at 24 h, 7 days, and 14 days post-injection using an IVIS^®^ Imaging System Lumina III (PerkinElmer, Waltham, MA, USA) under isoflurane anesthesia. After 7 and 14 days, the mice were sacrificed, and images of the organs and tumors were captured. The fluorescence radiance in the mice and tissues was quantified using the Living Image software program (PerkinElmer). Three different investigators were involved for each animal and were responsible for randomization, injection or measurement. During in vivo experiments, the mice were examined for signs of illness or physical activity. The mice were sacrificed if the length, width or height of the tumor was larger than 2 cm, or the body weight decreased by more than 20% of treatment-onset body weight.

### 4.13. mRNA Sequencing Analysis

Total RNA was extracted from UCMSCs (passage 6) and iMSCs (passage 13) using the TRIzol reagent (Invitrogen) according to the manufacturer’s protocol. The library construction, transcriptome sequencing, and subsequent analysis were conducted by OE Biotech Co., Ltd. (Shanghai, China). The libraries were sequenced on an Illumina Novaseq 6000 platform. Differential expression analysis was performed using DESeq2 (moderated estimation of fold change and dispersion for RNA-seq data with DESeq2), with significant differentially expressed genes (DEGs) identified based on a threshold q-value < 0.05 and |log₂ fold change| > 1. The Gene Ontology (GO) enrichment analysis of DEGs was performed using R (v3.2.0) based on the hypergeometric distribution to identify significantly enriched terms.

### 4.14. Statistical Analysis

Data are presented as mean ± standard error of the mean (SEM). For normally distributed data, statistical comparisons between two independent groups were performed using a *t*-test, while comparisons among multiple groups were conducted using ANOVA (GraphPad Prism 9.4.1). Non-normally distributed data were explored using the Mann–Whitney test. Statistical significance was defined as * *p* < 0.05, with additional levels of significance denoted as ** *p* < 0.01, *** *p* < 0.001, and **** *p* < 0.0001.

## Data Availability

The data supporting the findings of this study are presented in this article, including the Supplementary Materials, and they are available from the corresponding author upon reasonable request. The raw sequence data have been submitted to the NCBI Gene Expression Omnibus (GEO) datasets with accession number GSE276291.

## Supplementary Materials

The following supporting information can be downloaded at: https://www.mdpi.com/article/10.3390/ijms251910394/s1.
