# Supplementary material for: Mesenchymal Stem Cells Derived from Human Urine-Derived iPSCs Exhibit Low Immunogenicity and Reduced Immunomodulatory Profile
Source: Int J Mol Sci. 2024 Sep 27;25(19):10394. doi: 10.3390/ijms251910394 (PMC11476417; doi:10.3390/ijms251910394)
Supplement: Supplementary file 1 [file ijms-25-10394-s001.zip › ijms-3223149-supplementary.pdf]

Supplementary Materials

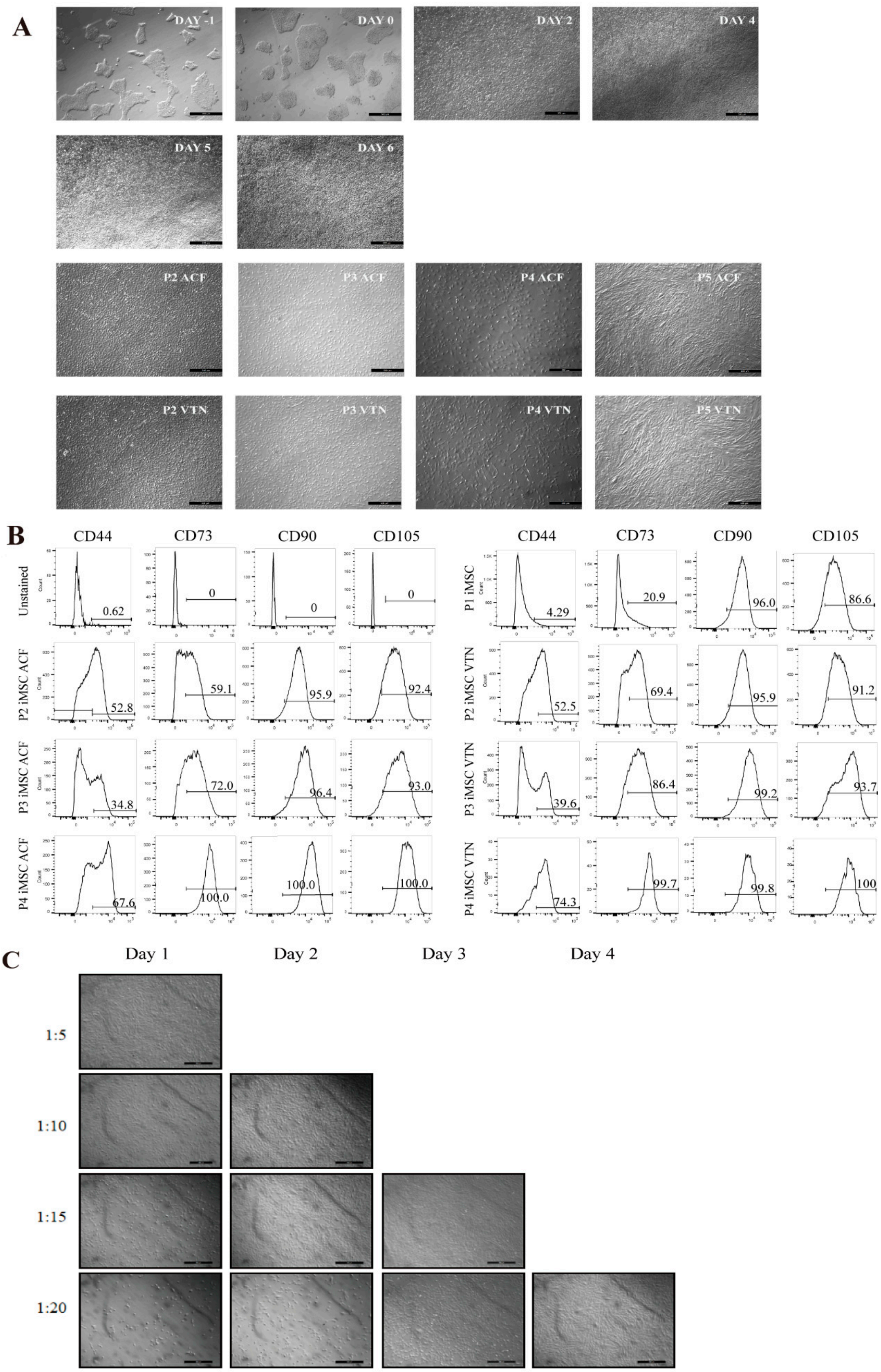

Figure S1. iMSC differentiation and characterization. (A) Morphological changes of iMSCs at various stages of differentiation (scale bar = 500 μm). (B) Flow cytometry analysis showing changes in cell surface markers on iMSCs at different differentiation stages. (C) Representative images demonstrating the proliferation of iMSCs at different passage ratios.

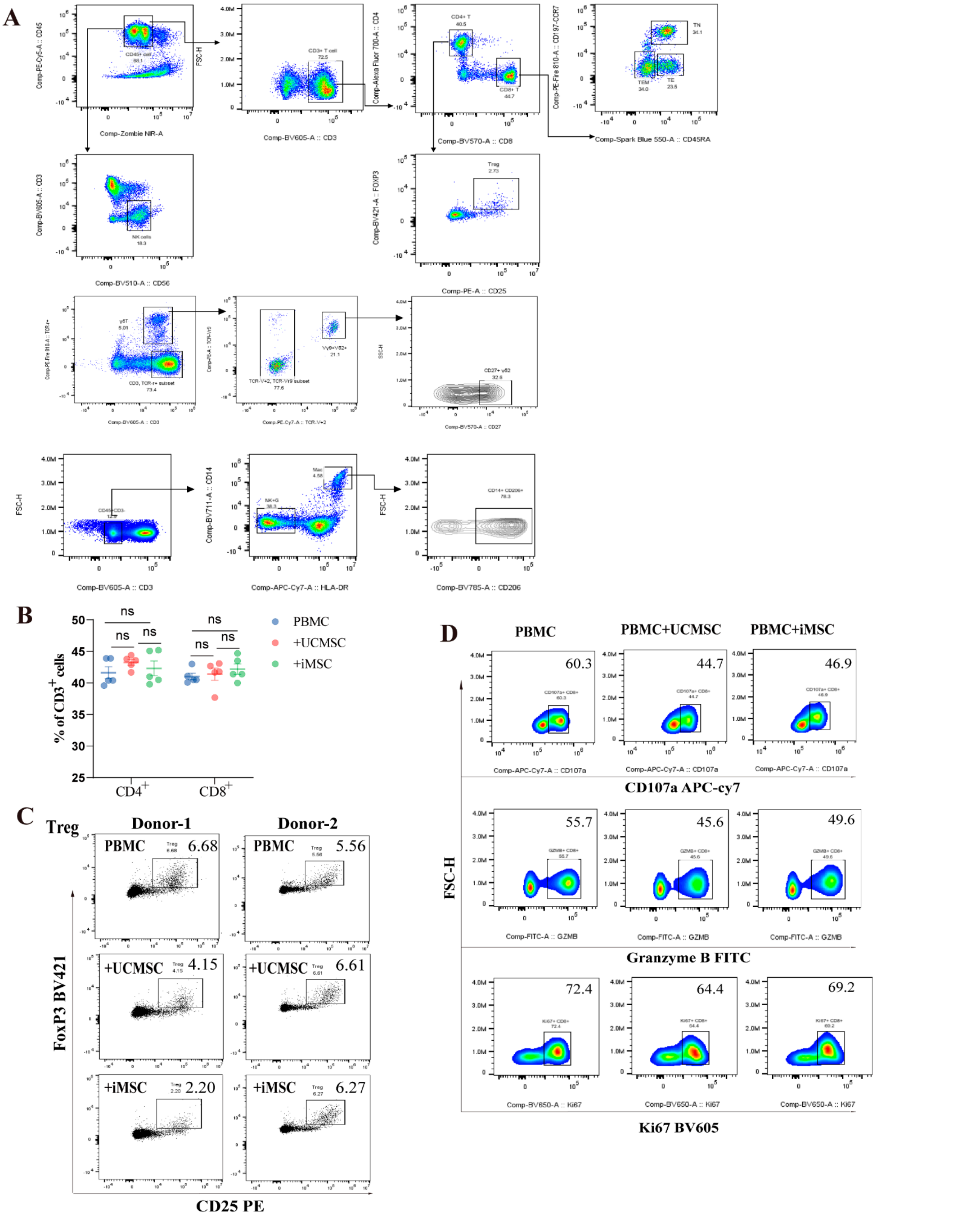

Figure S2. Assessment of iMSCs immunomodulatory ability. The differentiation status, proliferation, and activation of PBMCs, PBMCs co-cultured with UCMSCs (+UCMSC), or iMSCs (+iMSC) were analyzed by flow cytometry. (A) Gating strategy for PBMCs. (B) Percentages of $CD3^+CD4^+$ and $CD3^+CD8^+$ T cell populations across the three groups (mean ± SEM, n = 5, two-way ANOVA). (C) Identification of Treg cell subsets ($CD25^+FoxP3^+$) within $CD3^+CD4^+$ T cells. (D) Representative intracellular staining data for CD107a-APC-Cy7, granzyme B-FITC, and Ki67-BV605 within $CD3^+CD8^+$ T cell populations.

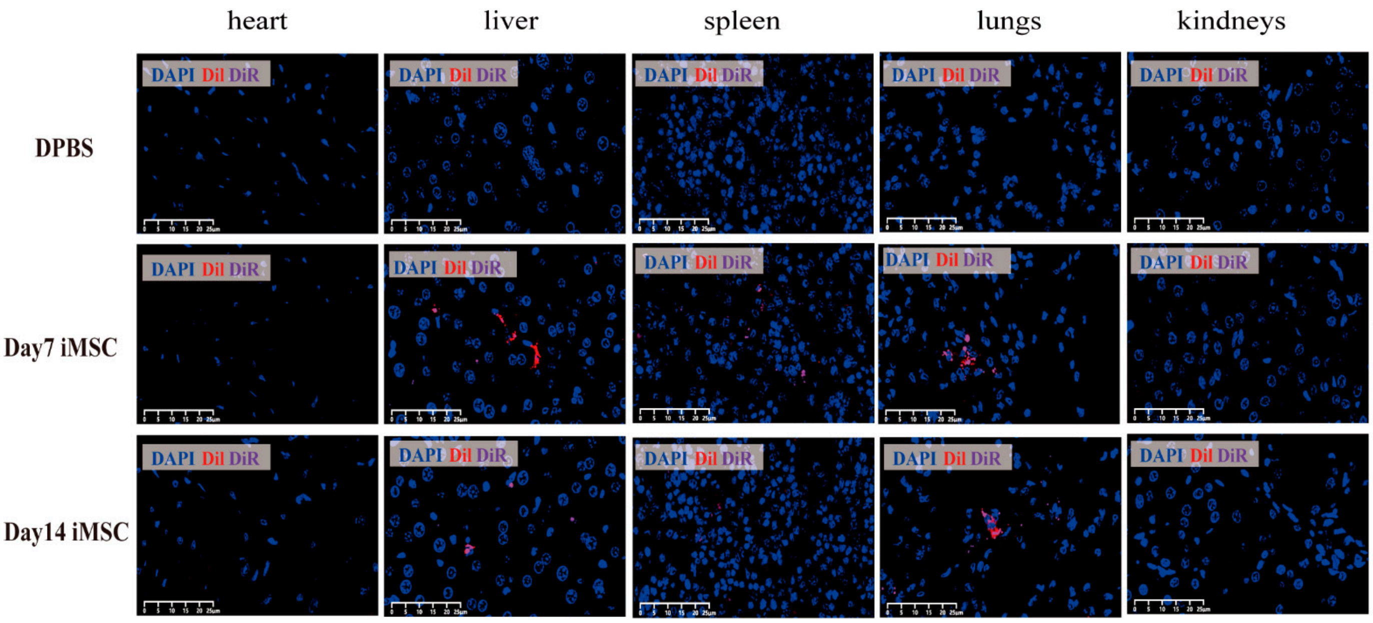

Figure S3. Representative images of DiI and DiR fluorescent signals in various organ sections detected via immunofluorescence analysis. Fluorescent signals were detected in different organ sections using immunofluorescence analysis (blue: DAPI, red: DiI, purple: DiR, scale bar = 25 μm).

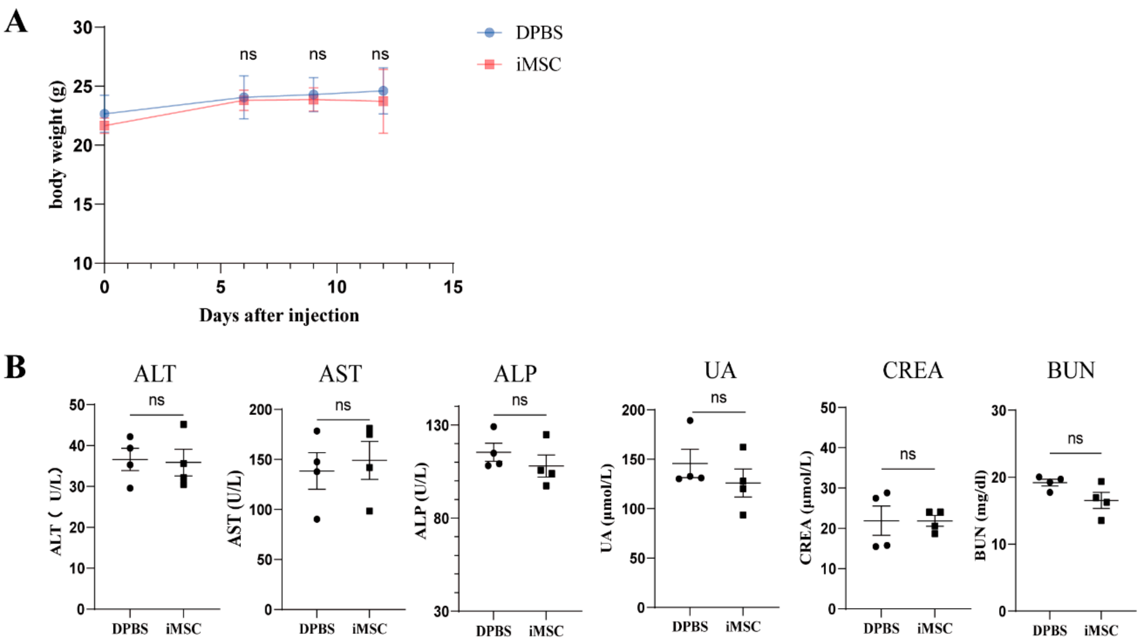

Figure S4. Evaluation of potential toxicity of iMSCs. (A) Body weight of NCG mice bearing A549 xenograft tumors was monitored for 12 days following iMSCs injection. (B) Effects of iMSCs treatment on blood biochemical indices (mean ± SEM, n = 4, two-sample *t*-test, ns, not significant). Measured indices include ALT (alanine transaminase), AST (aspartate aminotransferase), ALP (alkaline phosphatase), UA (uric acid), CREA (creatinine), and BUN (urea nitrogen).
